# Global trajectories of polygenic risk score research: a systematic bibliometric review of precision medicine, equity, and clinical translation

**DOI:** 10.3389/fmed.2026.1779659

**Published:** 2026-04-07

**Authors:** Fouad Bitar, Rana Zareef, Roukoz Abou-Karam, Georges Nemer, Fadi F. Bitar, Akl C. Fahed, Zahi Abdul Sater

**Affiliations:** 1 Cardiovascular Disease Initiative, Broad Institute of MIT and Harvard, Cambridge, MA, United States; 2 Division of Cardiology, Massachusetts General Hospital, Boston, MA, United States; 3 Children’s Heart Center, Department of Pediatrics and Adolescent Medicine, American University Beirut-Medical Center, Beirut, Lebanon; 4 College of Health and Life Sciences at Hamad Bin Khalifa University, Doha, Qatar; 5 Department of Medicine, Harvard Medical School, Boston, MA, United States; 6 College of Public Health, Phoenicia University, Mazraat El Daoudiyeh, Lebanon

**Keywords:** bibliometric, genetic risk score, polygenic risk score, polygenic score, precision medicine

## Abstract

**Introduction:**

Polygenic risk scores (PRS) have emerged as a central tool in genomic medicine, enabling risk prediction for common, complex diseases. Despite rapid methodological and clinical advances, concerns remain regarding the structural organization of PRS research, including geographic concentration, funding dominance, and limited global representation. A systematic, field-level assessment of PRS research evolution is needed to inform equitable and sustainable translation.

**Methods:**

We conducted a systematic bibliometric review of PRS research published between 1999 and 2024, using the Web of Science Core Collection to map publication growth, geographic and institutional contributions, funding patterns, collaboration networks, and thematic evolution.

**Results:**

The final dataset comprised 10,269 PRS-related publications across 2,185 sources, exhibiting a strong annual growth rate of 21.56%. Publication output accelerated markedly after 2017, reaching 1,580 articles in 2024. Logistic modeling demonstrated an excellent fit (*R*^2^ = 0.995), identifying a projected inflection point in 2026 and suggesting transition toward field maturation. While publication volume increased, mean citations per article declined over time, reflecting a shift from foundational studies to high-volume research output. PRS research was highly concentrated geographically and institutionally, with the United States, China, and the United Kingdom accounting for the majority of publications, and a small number of elite academic centers dominating output. International collaboration was substantial but unevenly distributed. Funding analysis revealed a pronounced core-periphery structure, with a limited set of public and philanthropic funders accounting for approximately one quarter of all funding acknowledgements. Thematic analyses showed a progression from foundational genetic concepts toward disease-specific risk prediction and clinical applications, particularly in neuropsychiatric, cardiometabolic, and oncological domains.

**Conclusion:**

PRS research has evolved into a mature, high-volume field with expanding clinical relevance, yet remains structurally concentrated in terms of geography, institutions, and funding. While bibliometric analyses do not directly reflect the ancestry composition of study populations, the observed concentration patterns highlight the importance of continued efforts toward broad collaboration, diversified funding landscapes, and transparent reporting practices to support globally representative and clinically robust implementation of PRS in precision medicine.

## 1 Introduction

Genomics has been reshaped over the past two decades by a convergence of forces: deeper biological insight, unprecedented data availability, the rise of population-scale biobanks, and a steep decline in sequencing costs (1, 2). Together, these shifts have moved genomic medicine beyond its early emphasis on rare, Mendelian disorders toward clinically relevant applications for common, complex diseases, where risk prediction, earlier detection, and prevention are central goals (3).

A cornerstone of this transition is the polygenic risk score (PRS), which quantifies an individual’s inherited susceptibility by aggregating the small effects of many genetic variants across the genome. The concept emerged alongside the first wave of genome-wide association studies (GWAS) in 2007 (4, 5) and was operationalized shortly thereafter, including in landmark work on schizophrenia that demonstrated how genome-wide risk propensity could be summarized at the individual level (6). Since then, PRS have become increasingly prominent in predicting risk for major conditions such as cardiovascular disease and cancer (7–10). PRS are generally constructed by combining effect size estimates from GWAS using established statistical methods such as clumping and thresholding, penalized regression techniques, or Bayesian shrinkage approaches. Improving risk stratification at the individual level, enabling more precise disease subtyping, identifying high-risk groups who may benefit from targeted screening or preventive strategies, and increasing statistical power in studies of gene–environment interactions and pharmacogenomics (3, 11, 12). Beyond prediction, PRS are now being explored for use in diagnostic refinement, enrichment and targeting strategies in clinical trials, and the prioritization of pathways and targets in drug discovery (10). Besides, in recent years, greater attention has been directed toward external validation, cross-population calibration, and integration of PRS into existing clinical risk models to facilitate their translation into clinical practice (1, 2).

Despite this promise, translation into equitable clinical practice remains constrained by two persistent barriers: representation and standardization. Most PRS methods and reference datasets have been developed using biobanks dominated by individuals of European ancestry, raising well-founded concerns about reduced predictive performance and potential downstream inequities when applied to underrepresented populations (13, 14). In parallel, variability in PRS construction, reporting, and validation practices complicates comparison across studies and slows consistent clinical adoption (15). These are not purely technical challenges. They are also products of how the field is organized including which populations are recruited into biobanks, where funding concentrates, which institutions lead model development, and how collaborative networks shape data access and validation practices (13, 14, 16). While bibliometric indicators do not directly capture the ancestry composition of study populations, they provide a systematic way to quantify these structural dynamics by mapping global contributions, funding sources, collaboration patterns, and thematic evolution (17, 18). This perspective complements technical evaluations by illuminating global patterns of productivity and collaboration, and by identifying areas where broader participation and capacity-building efforts may be warranted (19, 20).

Accordingly, we conducted a 25-year bibliometric review of PRS research to characterize global publication growth, geographic and institutional contributions, funding patterns, collaboration networks, and thematic trajectories, providing an evidence base to inform research investment, collaboration strategies, and discussions surrounding the global development and implementation of PRS (17, 18). To our knowledge, this is the first bibliometric analysis to comprehensively map PRS research across scientific productivity (including annual publication volume and country- and institution-level output), institutional leadership, funding sources, collaboration structures, and thematic evolution.

## 2 Materials and methods

### 2.1 Study design

This study is a systematic bibliometric review designed to map, quantify, and synthesize the global research landscape on polygenic risk scores (PRS). The review applies structured, reproducible search and selection procedures to identify relevant publications and uses established bibliometric techniques to analyze productivity, collaboration, funding structures, and thematic evolution across the field.

### 2.2 Review framework (PICOS adaptation)

- The review question and eligibility criteria were structured using an adapted PICOS framework appropriate for bibliometric and mapping reviews:
- Population: Scientific publications focused on polygenic risk scores.
- Intervention: Development, validation, application, and interpretation of PRS across diseases and populations.
- Comparators: Not applicable.
- Outcomes: Bibliometric indicators including publication volume, citation patterns, geographic and institutional contributions, international collaboration, funding sources, and thematic evolution.
- Study design: Original research articles indexed in the Web of Science Core Collection.

### 2.3 Source of data

A bibliometric review was conducted to analyze the PRS research landscape and its development over time. The Web of Science (WoS) database was selected as the primary data source due to its comprehensive coverage of scientific publications, extensive citation networks, structured bibliometric data, and indexing of high-impact journals, making it the preferred database for science mapping studies (21). Specifically, the Web of Science Core Collection, including the Science Citation Index Expanded (SCI-E), was used. To validate the robustness of the dataset, a sensitivity analysis using the same search strategy on PubMed showed a variation of less than 5%, based on comparisons of total publication counts and annual production trends, confirming the reliability of the WoS dataset.

### 2.4 Search strategy

A comprehensive literature search was conducted to identify publications related to PRS. The search strategy was developed by compiling a thorough list of PRS-related keywords from previous studies, reviews, meta-analyses, and the MEDLINE database. Boolean operators (AND, OR, NOT) were used to refine the search.

The search string employed was: TS = (“Polygenic risk score” OR “genetic risk score” OR “risk score, genetic” OR “risk score, polygenic” OR “polygenic risk” OR “polygenic score” OR “genetic profile” OR “polygenic prediction” OR “genetic risk prediction”).

Generic GWAS-only terms were intentionally excluded to ensure specificity to PRS-focused research rather than genome-wide association studies that did not involve risk score construction or application.

The inclusion of the term “genetic profile” was intended to capture early multivariant genetic risk aggregation approaches that predated standardized PRS terminology. During screening, studies not involving risk score construction or multivariant risk prediction were excluded to minimize inclusion of non-specific molecular profiling literature.

### 2.5 Eligibility criteria

The index period was January 1, 1999 through December 31, 2024. The extended time horizon was selected to capture early conceptual and methodological precursors to PRS research prior to the widespread adoption of GWAS terminology, as well as to comprehensively map the emergence and evolution of the field. We included original research articles, early access articles, and data papers. We excluded meeting abstracts, review articles, editorial material, proceeding papers, news items, letters, book chapters, retracted publications, and book reviews. All non-English articles were excluded. The exclusion of non-English publications may result in underrepresentation of research from some regions and is acknowledged as a limitation of the study.

### 2.6 Data management and selection process

The final list of references was extracted and analyzed using the Bibliometrix package, an R statistical software package for comprehensive science mapping analysis (18). All early access articles were de-duplicated to avoid double counting upon final publication indexing. The raw data exported from R were transformed into graphical and tabular formats to generate the following information: (a) annual scientific production; (b) journals in which researchers publish; (c) country-specific production; (d) authors’ countries and institutional affiliations; (e) collaboration patterns; (f) disciplines studied; and (g) funding agencies.

Analytical techniques included citation analysis, co-authorship and country collaboration network analysis, keyword co-occurrence mapping, and thematic evolution analysis. All analyses were conducted using Bibliometrix (version 5.2.1) implemented in R (version 2025.09.2).

Geographic and institutional analyses were based on author-reported affiliations as indexed in Web of Science metadata. Funding analyses were derived from funding acknowledgement fields. This study did not extract or analyze the ancestry composition of discovery or validation cohorts used in PRS construction, as such information is not systematically captured in bibliometric databases.

Ethics approval was not required as this study analyzed bibliographic metadata from publicly indexed literature without human participant data.

This systematic bibliometric review was conducted and reported in accordance with the PRISMA-ScR guidelines, where applicable (Supplementary Figure 1).

## 3 Results

### 3.1 Bibliometric dataset

The final bibliometric dataset comprised 10,269 PRS-related documents published between 1999 and 2024, indexed across 2,185 distinct sources, including journals and data publications (Table 1). The dataset demonstrated a strong annual growth rate of 21.56%, reflecting rapid expansion of PRS research over the study period. The average age of documents was 5.52 years, indicating that a substantial proportion of the literature is recent. Collectively, the documents cited 287,760 references, with an average of 30.09 citations per document, suggesting sustained scholarly impact.

**Table 1:** Descriptive characteristics of the PRS bibliometric dataset (1999–2024).

Description	Results
Main information	
Timespan	1999:2024
Sources (journals, books, etc.)	2,185
Documents	10,269
Annual growth rate %	21.56
Document average age	5.52
Average citations per doc	30.09
References	287,760
Document contents	
Keywords plus (ID)	14,968
Author’s keywords (DE)	14,720
Authors	
Authors	61,033
Authors of single-authored docs	130
Authors collaboration	
Single-authored docs	153
Co-authors per doc	13.1
International co-authorships %	44.63
Document types	
Article	10,210
Article; data paper	4
Article; early access	55

In terms of content structure, the dataset included 14,968 keywords plus terms and 14,720 author-provided keywords, reflecting a broad and diverse thematic landscape with close alignment between author-defined and indexer-assigned terminology. Authorship analysis identified 61,033 unique authors, with only 153 single-authored documents, corresponding to 130 individual authors, indicating that single-author publications were rare. Collaboration metrics revealed a high degree of co-authorship, with an average of 13.1 authors per document. Nearly half of all publications (44.63%) involved international co-authorship, underscoring the global and networked nature of PRS research.

### 3.2 Publication growth dynamics and logistic modeling

**Figure 1:**
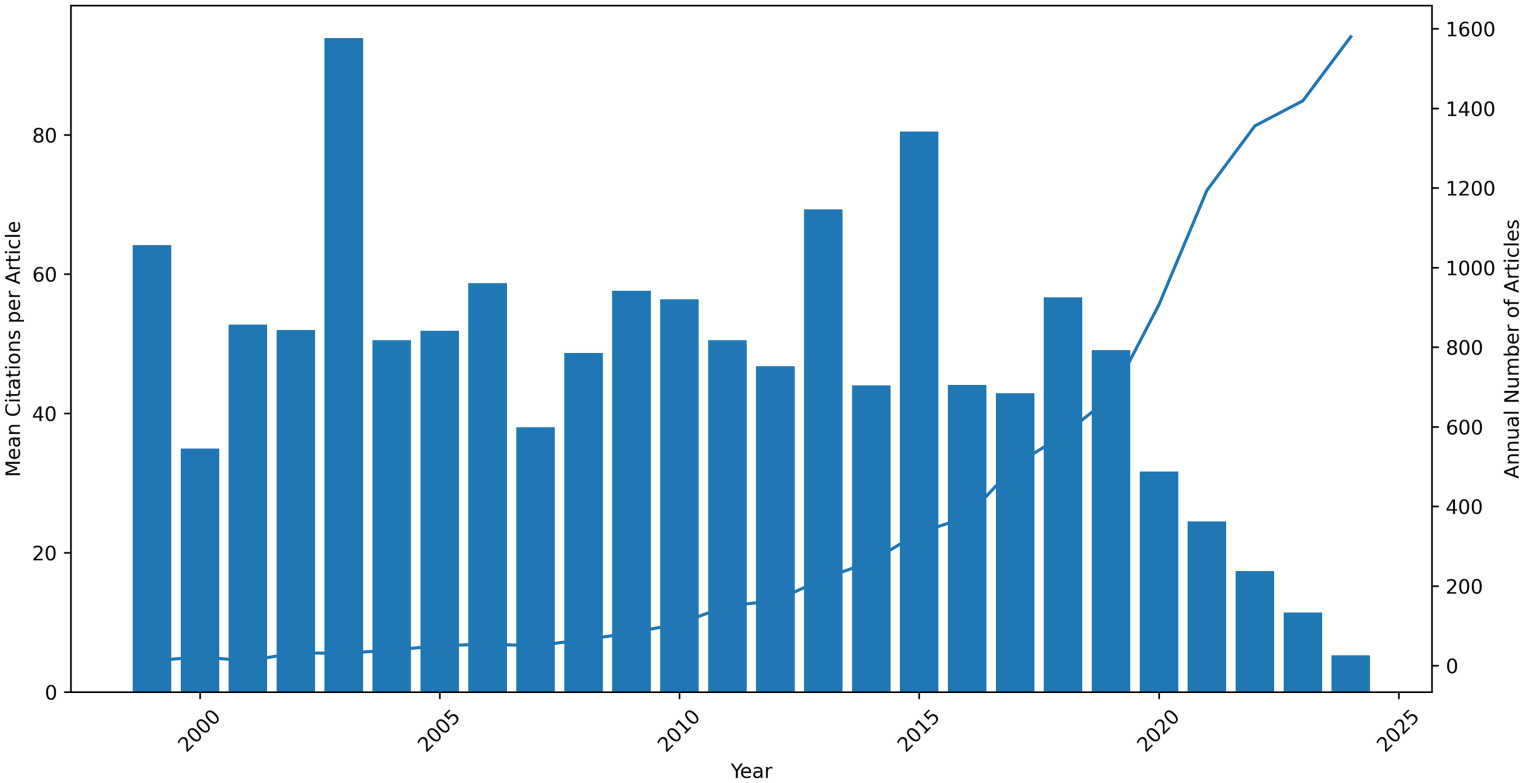
Annual scientific production and mean citations per year in PRS research (1999–2024). Annual number of PRS-related publications (blue line, right axis) and mean citations per article by year of publication (blue bars, left axis) indexed in the Web of Science database between 1999 and 2024. The figure demonstrates sustained growth in publication volume over time, particularly after 2017, alongside a progressive decline in mean citations per article in more recent publication years.

Across the study period, PRS research exhibited rapid and sustained growth in scientific output, culminating in 1,580 publications in 2024. Publication activity remained modest until the mid-2000s, followed by steady expansion through the early 2010s and a marked acceleration after 2017 (Figure 1). From 2020 onward, annual output exceeded 900 publications per year, indicating the transition of PRS from an emerging methodology to a high-volume research domain.

In contrast to publication growth, mean citation rates per article declined over time (Figure 1). Earlier publication cohorts consistently accrued higher average citation counts, with mean citations frequently exceeding 50 citations per article and peaking above 90 citations in the early years of PRS research. More recent publication cohorts, despite substantially higher output, exhibited markedly lower mean citation levels, falling below 20 citations per article in the most recent years. This divergence reflects a shift from a relatively small number of highly cited foundational studies toward a large volume of newer publications that have had less time to accumulate citations.

**Figure 2:**
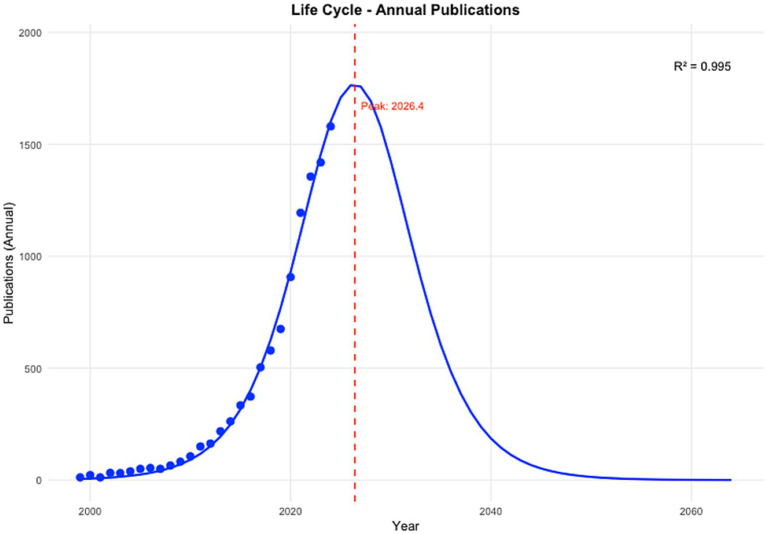
Logistic growth modeling of annual PRS scientific production (1999–2064). Observed annual counts of PRS-related publications indexed in Web of Science are shown as points, with the fitted logistic growth curve overlaid. The vertical dashed line denotes the estimated inflection point (2026.4), corresponding to the year of maximum growth rate in annual publication output. Values beyond 2024 represent model-based projections illustrating the expected deceleration in annual production as the field approaches saturation.

**Figure 3:**
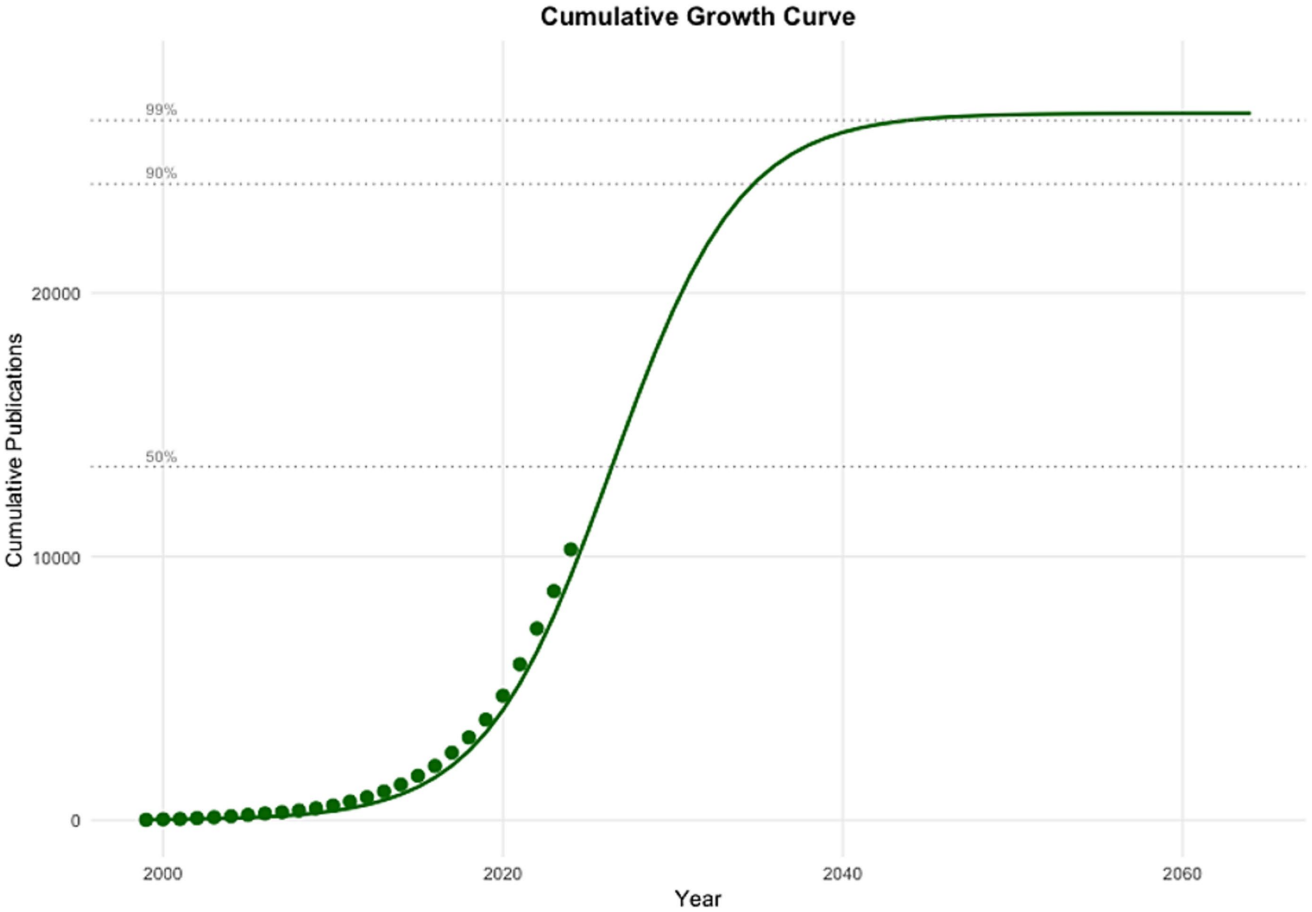
Logistic growth modeling of cumulative PRS scientific production (1999–2064). Observed cumulative PRS-related publications indexed in Web of Science are displayed alongside the fitted logistic growth curve. The model estimates a carrying capacity (*K*) of 26,818 cumulative publications, toward which projected output converges asymptotically. Forecasted values beyond 2024 illustrate the transition from accelerated growth to a saturation phase.

Logistic growth modeling of annual PRS publication output demonstrated a highly structured and non-linear growth trajectory, characterized by an initial emergence phase, a prolonged period of acceleration, and a projected transition toward saturation (Figure 2). The logistic model showed an excellent fit to the observed data (*R*^2^ = 0.995; RMSE = 35.4), explaining approximately 99.5% of the variance in annual publication counts. The model identified the inflection point (*t*_0_), corresponding to the year of maximum annual growth rate at 2026.4, with a predicted peak annual output of approximately 1,769 publications. Observed publication counts up to 2024 closely followed the fitted trajectory, with annual production increasing from 12 publications in 1999 to 1,580 publications in 2024, consistent with the steep ascent predicted during the expansion phase. Based on model projections, cumulative PRS publications reached 10% of total saturation by 2018, and are expected to reach 90% by approximately 2034.7, and 99% by 2043.8, after which annual publication rates are projected to decline progressively (Figure 3).

### 3.3 Source concentration and journal quality

**Figure 4:**
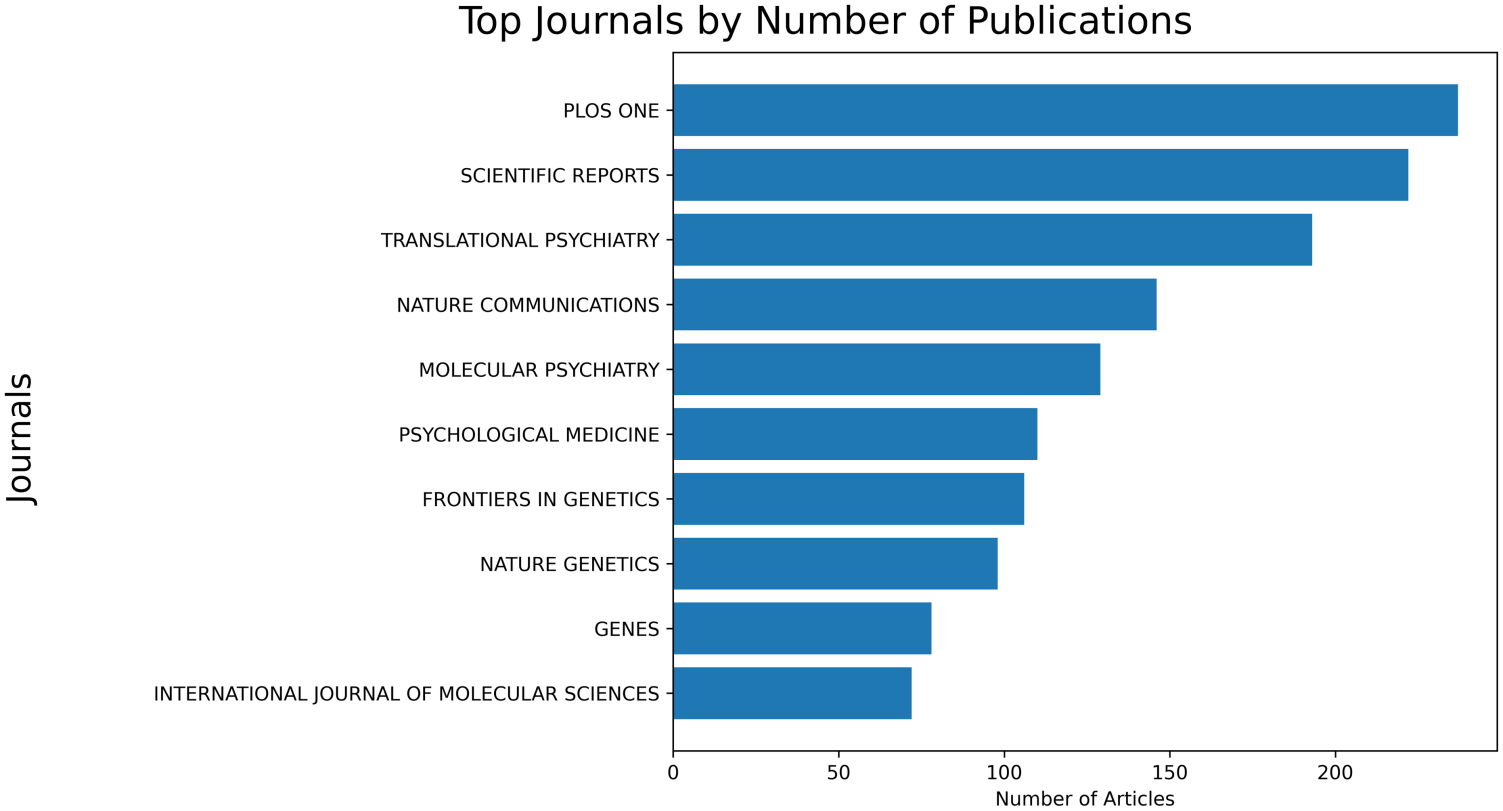
Most productive journals publishing PRS research. Top journals by number of PRS-related publications indexed in Web of Science (1999–2024).

PRS-related publications were distributed across a wide range of journals, with the most productive sources spanning multidisciplinary science, genetics, psychiatry, and clinical medicine (Figure 4). Among the leading outlets, PLoS One (*n* = 237) and Scientific Reports (*n* = 222) accounted for the highest number of publications, followed by Translational Psychiatry (*n* = 193), Nature Communications (*n* = 146), and Molecular Psychiatry (*n* = 129). Additional high-volume contributors included Psychological Medicine (*n* = 110), Frontiers in Genetics (*n* = 106), Nature Genetics (*n* = 98), Genes (*n* = 78), and the International Journal of Molecular Sciences (*n* = 72). Assessment of journal impact using Journal Citation Reports (JCR) quartiles showed that PRS research is predominantly published in high-ranked journals. Of the top 10 most productive sources, six were classified as Q1 journals (including Nature Communications, Nature Genetics, Molecular Psychiatry, Translational Psychiatry, Psychological Medicine, and International Journal of Molecular Sciences), while three were Q2 journals (Scientific Reports, Frontiers in Genetics, and PLoS One).

### 3.4 Geographic and institutional distribution of PRS research

**Figure 5:**
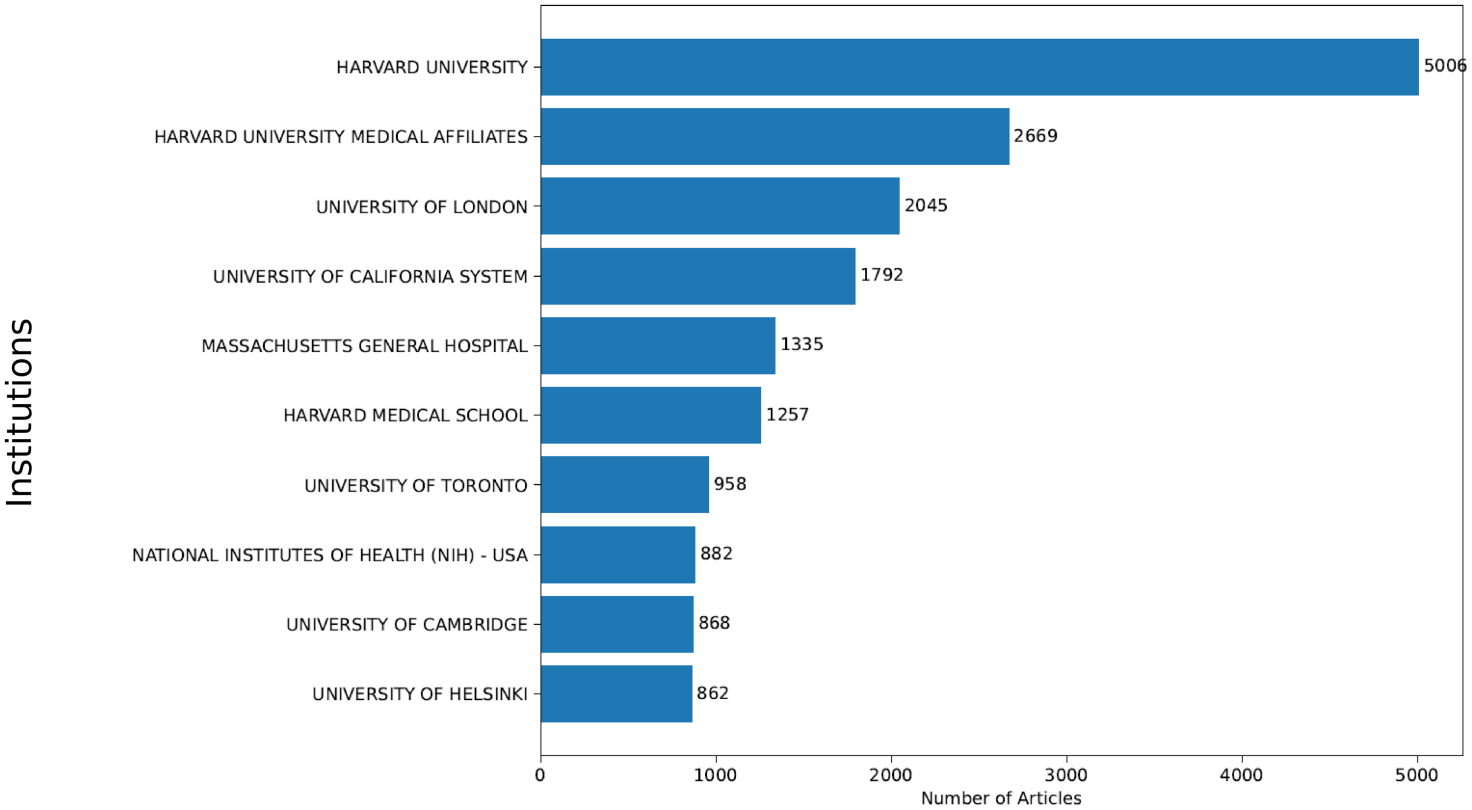
Most productive institutions in PRS research (1999–2024). Top academic and research institutions by number of PRS-related publications indexed in the Web of Science database between 1999 and 2024. Bar length represents total publication output per institution.

**Figure 6:**
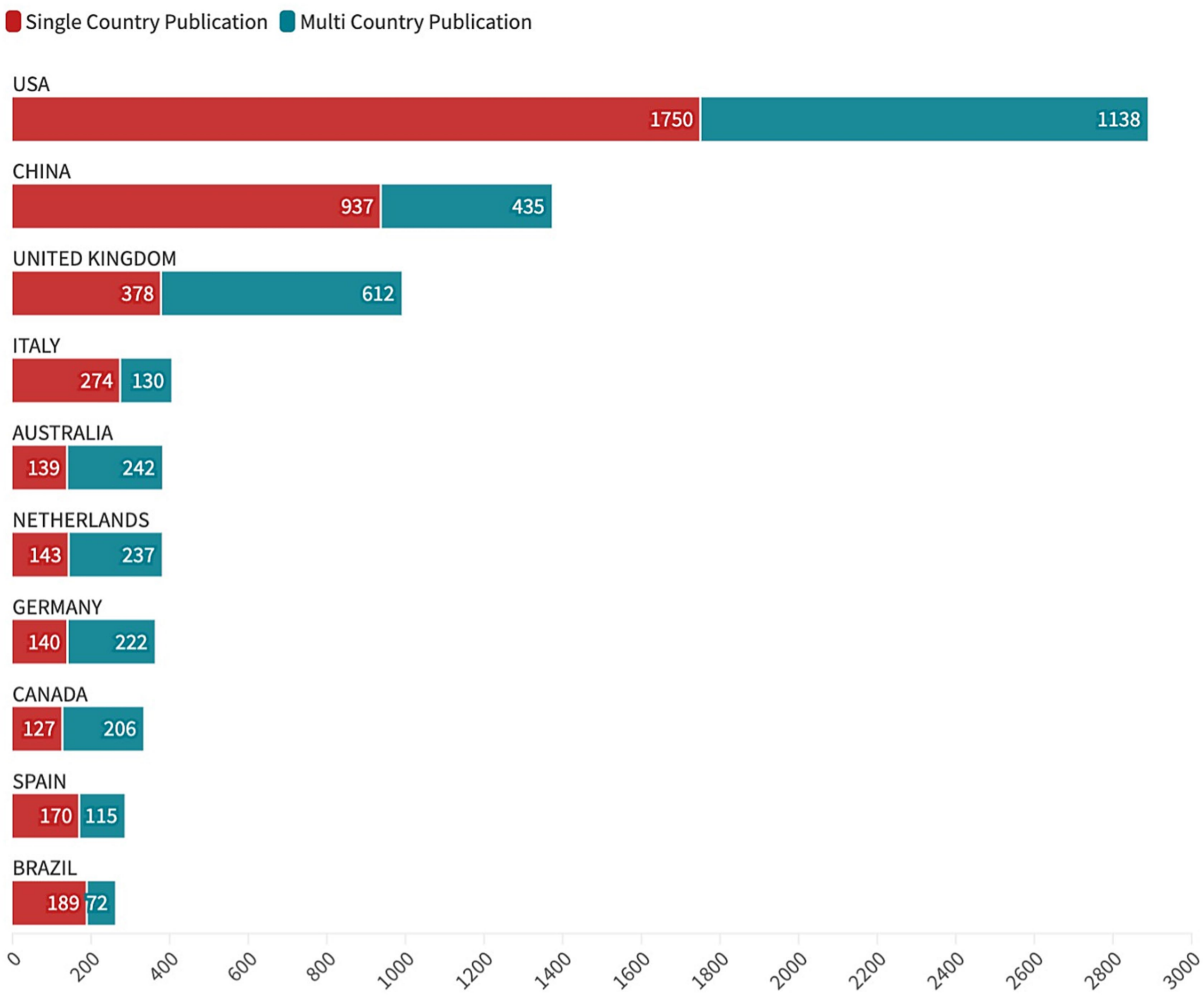
Corresponding authors’ countries and international collaboration patterns in PRS research. Distribution of PRS-related publications by corresponding authors’ countries, stratified by single-country publications (SCP) and multiple-country publications (MCP), indexed in Web of Science from 1999 to 2024. Bar length reflects total publication count, with color segments indicating the relative contribution of domestic and internationally collaborative research.

PRS research output was highly concentrated among a small number of countries and institutions, with marked differences in both productivity and international collaboration patterns (Figures 5, 6). The United States was the most prolific contributor, accounting for 2,888 publications, followed by China (1,372 publications) and the United Kingdom (990 publications). Additional major contributors included Italy, Australia, the Netherlands, Germany, and Canada, each producing between approximately 330 and 400 publications, reflecting a geographically diverse but uneven global research landscape.

Analysis of corresponding authorship revealed substantial variation in international collaboration, as measured by the proportion of multiple-country publications (MCP). Several countries exhibited high levels of international engagement, including Finland (74.0%), Norway (66.7%), Sweden (65.1%), Australia (63.5%), the Netherlands (62.4%), Canada (61.9%), the United Kingdom (61.8%), and Germany (61.3%), indicating that the majority of their PRS research output involved cross-border collaboration. In contrast, the United States (39.4%) and China (31.7%), despite being the most productive countries overall, demonstrated lower MCP proportions, reflecting a larger share of single-country publications (SCP). Nevertheless, due to their high publication volume, both countries contributed substantial absolute numbers of internationally collaborative studies.

Institutional productivity mirrored national patterns, with Harvard University (5,006 publications) and Harvard University-affiliated medical institutions (2,669 publications) representing the most prolific contributors globally. Other leading institutions included the University of London, the University of California system, Massachusetts General Hospital, Harvard Medical School, the University of Toronto, the U.S. National Institutes of Health, the University of Cambridge, and the University of Helsinki, underscoring the dominance of large, research-intensive academic and medical centers in PRS scholarship.

### 3.5 International collaboration network in PRS research

**Figure 7:**
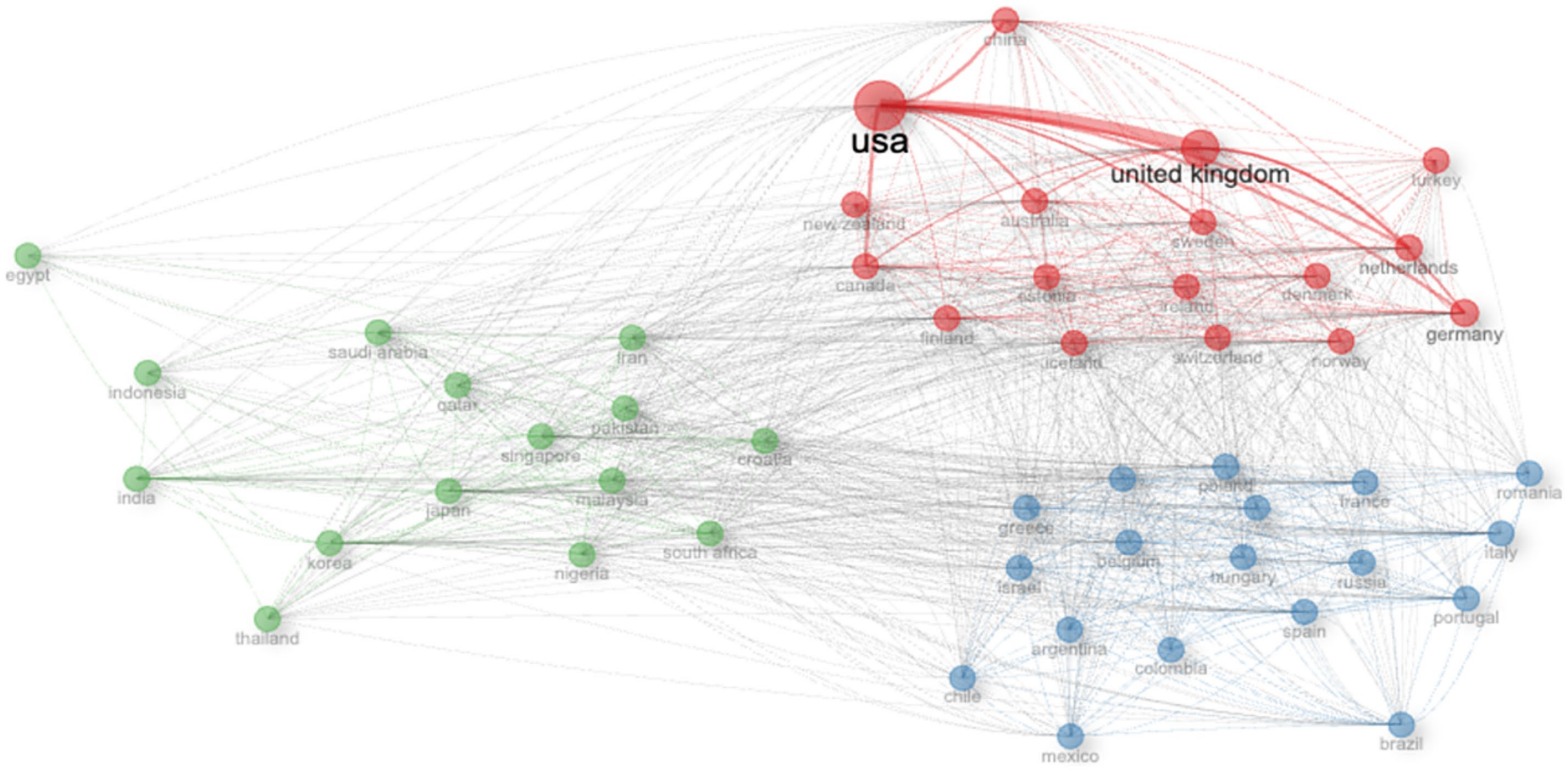
International co-authorship network of PRS research. Network visualization of international co-authorships in PRS-related publications indexed in Web of Science (1999–2024). Nodes represent countries, with node size proportional to collaboration intensity. Edges represent co-authorship links between countries, with thickness corresponding to link strength. Colors indicate collaboration communities identified using the Louvain community detection algorithm.

Country-level co-authorship network analysis revealed a highly structured international collaboration landscape, characterized by distinct collaboration communities and strong centralization around a limited number of countries (Figure 7). The network exhibited a clear community-based structure, with three major collaboration clusters identified using the Louvain community detection algorithm. Countries within each cluster demonstrated stronger internal collaboration ties relative to cross-cluster connections, indicating preferential collaboration patterns.

The largest and most interconnected cluster was centered on the United States, which occupied a dominant and highly central position in the network. This cluster included other major research-intensive countries such as the United Kingdom, China, Canada, Australia, Germany, and several Northern and Western European countries, reflecting dense and frequent co-authorship relationships among these nations. A second cluster comprised predominantly Asian, Middle Eastern, and African countries, with India and Japan emerging as central nodes, alongside countries such as Saudi Arabia, Qatar, Singapore, Malaysia, and South Africa. A third cluster consisted mainly of European and South American countries, including France, Italy, Spain, Poland, and Brazil, which formed a cohesive collaboration community distinct from the other clusters.

Node size distribution indicated substantial heterogeneity in collaboration intensity, with a small number of countries exhibiting disproportionately high connectivity, such as USA and UK. While cross-cluster collaborations were present, they were generally weaker than within-cluster ties, underscoring the modular structure of the global PRS collaboration network.

### 3.6 Thematic structure of PRS research

**Figure 8:**
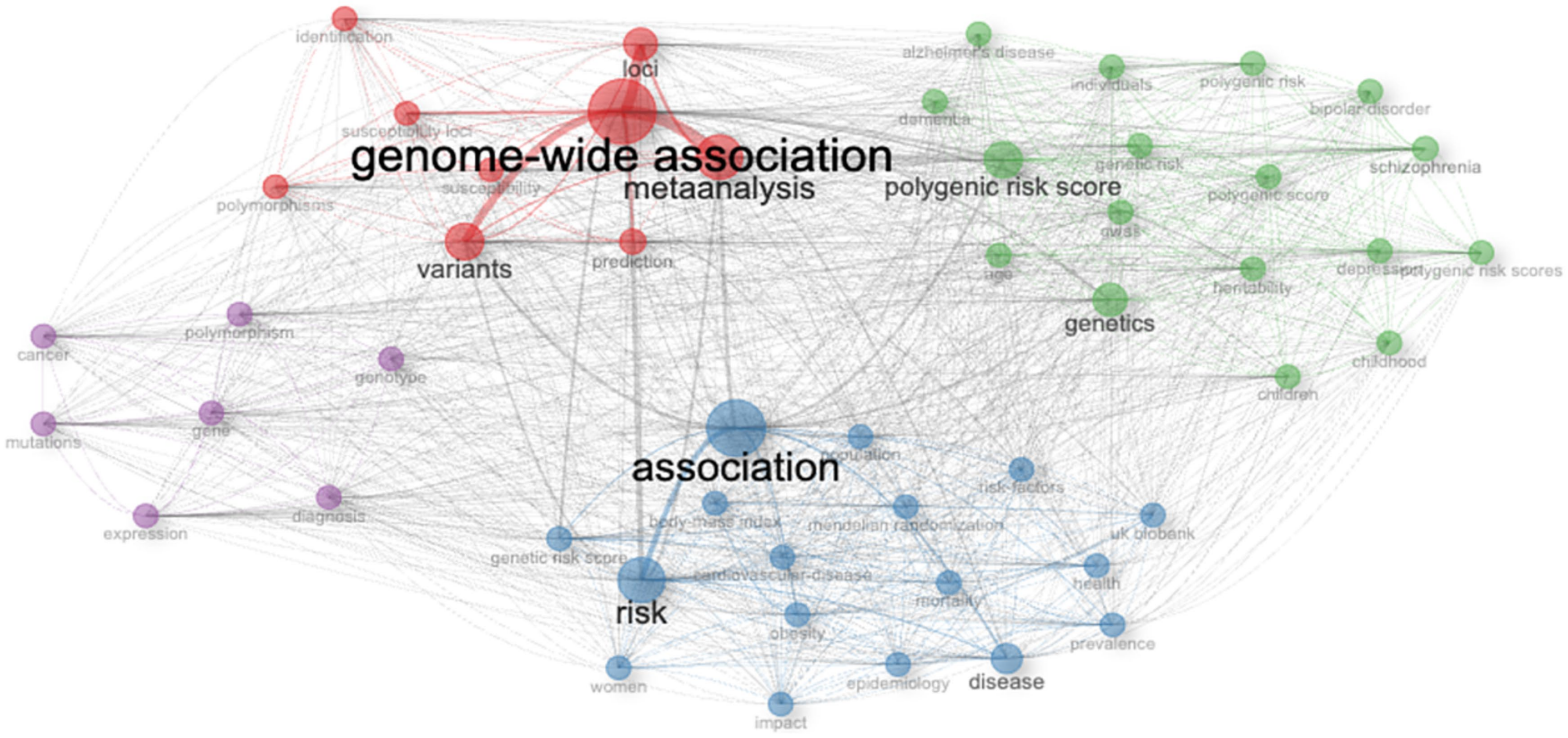
Keyword co-occurrence network of PRS research themes. Network visualization of keyword co-occurrence in PRS-related publications indexed in Web of Science (1999–2024). Nodes represent keywords, with node size proportional to frequency of occurrence. Edges represent co-occurrence relationships, with thickness corresponding to connection strength. Colors denote thematic clusters identified using the Louvain community detection algorithm.

Keyword co-occurrence network analysis revealed a structured and multi-cluster thematic organization of PRS research (Figure 8). Four dominant thematic communities were identified reflecting distinct but interconnected research foci. The largest and most central cluster was anchored by genome-wide association loci variants and meta-analysis indicating the foundational role of GWAS methodologies in structuring the PRS literature. This cluster showed dense internal connectivity and strong links to other thematic areas underscoring its function as a methodological backbone of the field.

A second major cluster centered on polygenic risk score, genetics, and heritability, with strong connections to disease-specific terms such as schizophrenia, bipolar disorder, Alzheimer’s disease, and depression, reflecting the application of PRS frameworks to neuropsychiatric and neurological conditions. A third cluster was organized around association, risk, and disease, incorporating epidemiological and clinical concepts including risk factors, prevalence, morbidity, and population-based resources such as the UK Biobank, highlighting the integration of PRS into population health and disease risk assessment.

A smaller, distinct cluster focused on cancer-related and molecular genetic terms, including mutations, genotype, expression, and polymorphism, representing disease-specific and mechanistic research streams. Highly connected nodes such as association, risk, genome-wide association, and polygenic risk score served as bridges between clusters, indicating substantial thematic overlap and reinforcing the interdisciplinary nature of PRS research across genetic discovery, risk prediction, and clinical application.

### 3.7 Thematic evolution of PRS research

Trend topic analysis based on merged author and index keywords revealed a clear temporal progression in the thematic focus of PRS research over the study period (Table 2). Early research activity (approximately 1999–2006) was dominated by foundational genetic and molecular biology concepts, including polymorphisms, susceptibility loci, single nucleotide polymorphisms, DNA, loci, and population-level variation, reflecting the methodological groundwork preceding widespread PRS implementation. These terms exhibited early and sustained presence, indicating their role as core building blocks of the field.

**Table 2:** Temporal evolution of dominant research themes in PRS literature (1999–2024).

Term	Frequency	Year (Q1)	Year (median)	Year (Q3)
Genome-wide association	1,825	2017	2020	2022
Association	1,716	2019	2021	2023
Risk	1,299	2018	2021	2023
Meta-analysis	1,119	2018	2021	2023
Polygenic risk score	1,029	2021	2022	2023
Variants	927	2017	2020	2022
Loci	772	2017	2020	2022
Health	439	2020	2022	2023
Polymorphisms	382	2015	2019	2022
Polygenic risk scores	360	2021	2022	2023
Polymorphism	332	2014	2018	2022
Susceptibility loci	306	2017	2019	2021
Body-mass index	287	2017	2019	2022
UK Biobank	220	2022	2023	2024
Common variants	197	2015	2018	2021
Genetic susceptibility	128	2020	2023	2024
DNA	115	2012	2017	2021

During the mid-development phase (approximately 2006–2014), the thematic landscape expanded to include genetic risk prediction, genome-wide association-related terminology, and laboratory and analytical techniques such as microarrays, microsatellite instability, immunohistochemistry, and promoter analysis. This period was characterized by increasing keyword frequency and diversification, consistent with the transition from candidate-gene approaches toward genome-wide and integrative analytical frameworks.

In more recent years (approximately 2014 onward), PRS research showed a pronounced shift toward application-oriented and disease-relevant themes, including tumor suppressor genes, loss of heterozygosity, short tandem repeats, p53-related pathways, body-mass index, and disease-specific risk modeling. Concurrently, population-scale resources and computational approaches—such as biobanks, polygenic risk scores, and deep learning—emerged as prominent and increasingly frequent topics. The sustained growth and increasing frequency of these terms indicate consolidation of PRS as a central analytic paradigm and its expansion across diverse biomedical and clinical domains.

### 3.8 Funding landscape and evolution of PRS research

Funding analysis based on the Web of Science funding agency field (FU) revealed a highly concentrated funding structure underlying PRS research. A limited number of public and philanthropic agencies accounted for a substantial share of funding acknowledgements across the corpus. The most frequently cited funders were the U.S. National Institutes of Health (NIH), the UK Medical Research Council (MRC), and the Wellcome Trust, followed by national research agencies and disease-specific charities, including the Canadian Institutes of Health Research, the Sigrid Jusélius Foundation, the Novo Nordisk Foundation, the Swedish Research Council, Cancer Research UK, and the British Heart Foundation (Table 3). Funding concentration analysis showed that the top five funding agencies accounted for approximately one-fifth of all funding acknowledgements, with the top 10 funders together accounting for approximately one quarter, indicating a pronounced core-periphery structure in PRS research financing. This pattern reflects reliance on a small set of dominant public and philanthropic funders supporting a large and expanding body of PRS-related publications.

**Table 3:** Leading funding agencies supporting polygenic risk score research (1999–2024).

Rank	Funding agency	Acknowledged papers (*n*)	% of all papers	% of funded papers
1	U.S. National Institutes of Health (NIH)	2,938	28.18	32.86
2	UK Medical Research Council (MRC)	1,688	16.19	18.88
3	EUROPEAN Union/European Research Council (EU/ERC)	798	7.65	8.92
4	NATIONAL Natural Science Foundation of China (NSFC)	689	6.61	7.71
5	Wellcome Trust	643	6.17	7.19
6	UKRI/NIHR (UK)	356	3.41	3.98
7	CANADIAN Institutes of Health Research (CIHR)	340	3.26	3.8
8	Swedish Research Council	278	2.67	3.11
9	Cancer Research UK	262	2.51	2.93
10	Novo Nordisk Foundation	214	2.05	2.39
11	Academy of Finland	209	2	2.34
12	British Heart Foundation	186	1.78	2.08
13	National Health and Medical Research Council	172	1.65	1.92
14	German Research Foundation	160	1.53	1.79
15	National Institute for Health Research	157	1.51	1.76
16	Swedish Heart-Lung Foundation	147	1.41	1.64
17	Alexander von Humboldt Foundation	130	1.25	1.45
18	National Science Foundation	129	1.24	1.44
19	Australian Research Council	122	1.17	1.36
20	National Institute for Health Research (NIHR)	120	1.15	1.34

Summary of the top funding agencies acknowledged in PRS-related publications indexed in the Web of Science database between 1999 and 2024. The table reports the number and proportion of papers acknowledging each funder, illustrating the concentration of research support among a limited set of public and philanthropic funding bodies.

Temporal analysis of funding acknowledgements demonstrated a marked increase in funding intensity over time, closely mirroring the rapid expansion of PRS publication output (Table 4). Prior to 2007, funding acknowledgements were sparse and fragmented. From approximately 2010 onward, major public and philanthropic funders showed sustained growth in funding presence, particularly the NIH, UK MRC, and Wellcome Trust. Funding acknowledgements peaked during the 2020–2023 period, coinciding with the highest annual publication volumes observed in the corpus, before declining slightly in the most recent year, consistent with partial indexing of recent publications.

**Table 4:** Temporal distribution of funding acknowledgements for major PRS funding agencies (1999–2024).

Funder	1999–2006	2007–2011	2012–2016	2017–2020	2021–2024
U.S. National Institutes of Health (NIH)	0	74	417	863	1,549
UK Medical Research Council (MRC)	3	15	227	526	908
Wellcome Trust	1	11	95	225	303
European Union/European Research Council (EU/ERC)	0	7	85	248	445
National Natural Science Foundation of China (NSFC)	0	2	47	132	489
Canadian Institutes of Health Research (CIHR)	0	1	45	96	190

Number of PRS-related publications acknowledging selected major funding agencies across successive time periods, showing the evolution and intensification of funding support in parallel with the expansion of PRS research output.

Overall, these trends indicate that the acceleration of PRS research has been accompanied by increasing engagement from a small number of large, well-established funding bodies, reinforcing the central role of public and philanthropic investment in shaping the field’s growth trajectory.

## 4 Discussion

**Figure 9:**
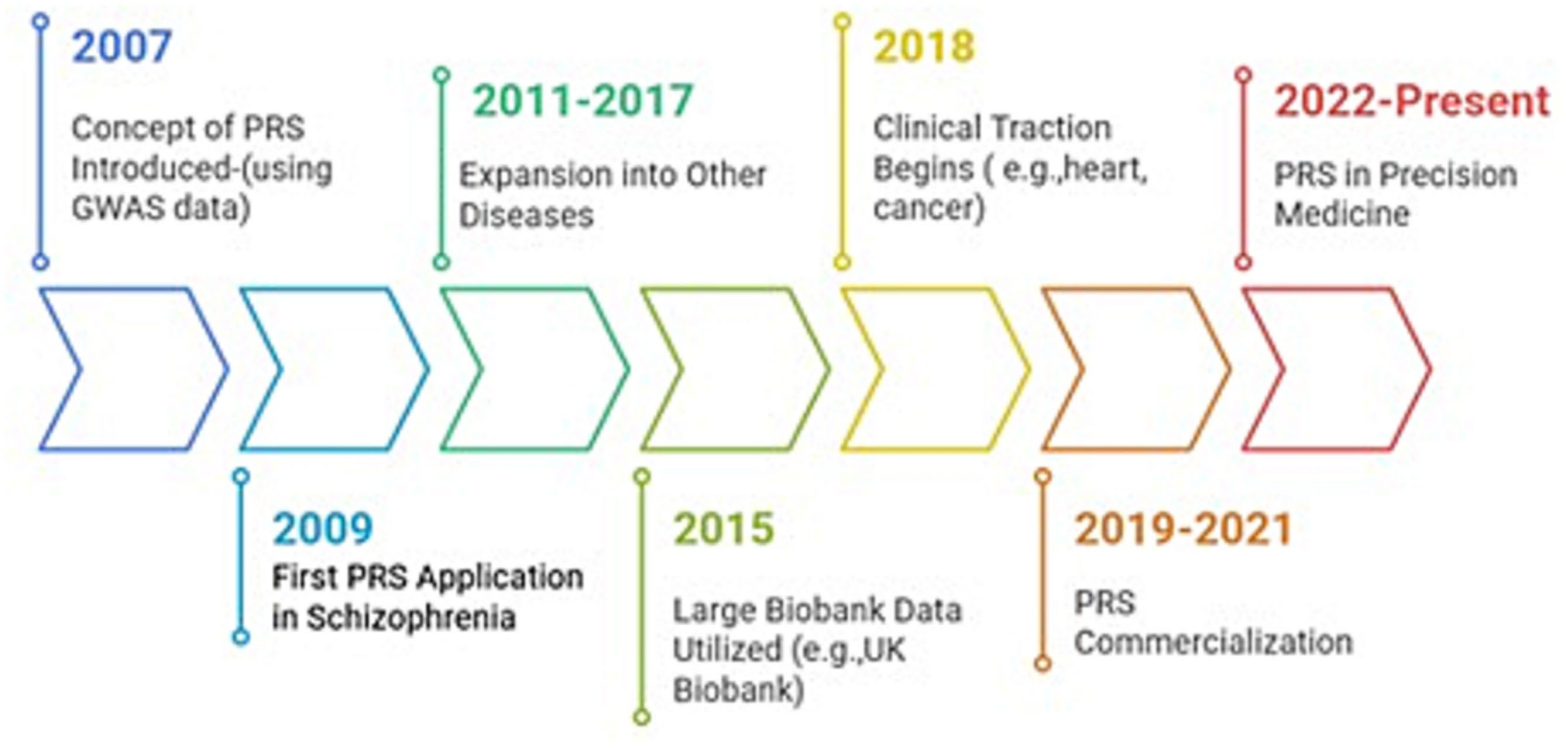
Important time frames in PRS research development. Flow diagram depicting the development of polygenic risk scores across time.

This bibliometric analysis provides a comprehensive, longitudinal perspective on the evolution of polygenic risk score (PRS) research over a 25-year period, revealing a field that has transitioned from methodological experimentation to large-scale application, while remaining structurally concentrated in terms of geography, institutions, and funding. The first application in schizophrenia appeared in 2009 (6), later extending to include cardiometabolic diseases and cancer (8, 9, 12). The utilization of large biobanks, such as the UK Biobank in 2015, marked a turning point in PRS modeling (2). Clinical application commenced in 2018 (2, 3, 10, 12) followed by commercialization in 2019 (10). By 2022, healthcare systems began integrating PRS into their preventive screening programs and personalized risk assessment (Figure 9) (3, 10).

### 4.1 From conceptual emergence to field maturation

The temporal trajectory of PRS research reflects a classic pattern of scientific field development. Early work, emerging in the late 2000s, was closely tied to genome-wide association studies and focused on demonstrating the feasibility of aggregating common genetic variants to capture disease risk (5, 6). This foundational phase was characterized by a small number of highly cited studies, consistent with the elevated mean citation rates observed for early publication cohorts (22). As the field progressed into the 2010s, the availability of large biobank datasets, including population-scale resources such as the UK Biobank, enabled rapid expansion of PRS development across multiple phenotypes and diseases, accelerating both publication volume and thematic diversification (2, 8).

Logistic growth modeling confirmed that PRS research has followed a structured, non-linear expansion, with a prolonged acceleration phase and a projected inflection point around 2026. This finding suggests that PRS has moved beyond a purely exploratory stage and is entering a phase of consolidation and refinement (10). The divergence observed between rapidly increasing publication volume and declining mean citation rates per article is consistent with this transition, reflecting the shift from a small number of seminal contributions toward a high-throughput research ecosystem in which incremental advances predominate (17, 22). Such dynamics are characteristic of maturing methodological fields and underscore the importance of synthesis, standardization, and translational evaluation (15).

### 4.2 Structural concentration and global inequities

Despite its global scientific relevance, PRS research remains highly concentrated (13, 14). Geographic and institutional analyses revealed that a small number of high-income countries—particularly the United States, China, and the United Kingdom dominate research output, while leading academic and medical centers account for a disproportionate share of publications. This concentration is reinforced by collaboration patterns: while many European and smaller high-income countries exhibit high proportions of international co-authorship, the most productive countries rely more heavily on domestic research capacity, reflecting differences in scale, infrastructure, and funding ecosystems (23, 24).

The international collaboration network further illustrates the modular structure of PRS research, with dense collaboration clusters centered on a limited number of scientific hubs (25, 26). Although cross-cluster ties exist, collaboration remains preferential and uneven, suggesting that access to PRS research networks is shaped by institutional prestige, historical partnerships, and resource availability (23). These patterns highlight asymmetries in research leadership and collaboration structures, underscoring the importance of examining how global research capacity is distributed across institutions and regions (14).

### 4.3 Funding concentration and its implications

Funding analysis provides additional insight into the structural drivers of PRS research (17, 18). A small group of public and philanthropic funding agencies account for a substantial share of funding acknowledgements. The observed core-periphery funding structure mirrors patterns of publication and institutional concentration, highlighting the central role of a limited number of funders in shaping research agendas, methodological priorities, and translational pathways (13).

The temporal evolution of funding acknowledgements closely parallels publication growth (17, 18), indicating that the rapid expansion of PRS research has been underwritten by sustained investment from established funding bodies. While such investment has enabled methodological advances and large-scale studies, continued concentration of funding within a limited set of research ecosystems may influence the geographic distribution of research activity and infrastructure development (13, 14). Without deliberate efforts to broaden funding access and research participation, the global applicability of PRS may remain constrained.

### 4.4 Thematic shifts and translational orientation

Thematic analyses reveal a clear evolution from foundational genetic concepts toward application-oriented research (3, 10). Early emphasis on polymorphisms, loci, and molecular techniques gave way to genome-wide approaches and genetic risk prediction (5, 6), followed by increasing focus on disease-specific risk modeling, population health applications, and computational methods (8, 9). The growing prominence of biobanks, polygenic risk scores, and machine learning reflects the integration of PRS into large-scale epidemiological and clinical research frameworks (2, 12).

Notably, neuropsychiatric disorders have played a central role in shaping PRS research, likely reflecting the complex genetic architecture of these conditions and the absence of definitive biomarkers (6, 10). More recently, cardiometabolic and oncological applications have expanded rapidly, signaling a shift toward preventive and precision medicine use cases (8, 10). This progression aligns with the increasing interest in PRS as a tool for risk stratification, early intervention, and clinical decision support (3, 12).

### 4.5 Implications for equity and future directions

Taken together, these findings indicate that while PRS research has expanded rapidly in scale and scope, its bibliometric footprint remains concentrated within a limited set of countries, institutions, and funding bodies (3, 10). Although bibliometric indicators do not directly capture the ancestry composition of study populations or the performance of PRS across diverse groups, the observed concentration patterns underscore the importance of continued efforts to broaden global participation in PRS research and infrastructure development (13, 14). Expanding collaborative networks, diversifying funding streams, and strengthening research capacity across regions may support more inclusive and globally relevant innovation in polygenic risk prediction (13, 14, 20).

Future research should prioritize not only methodological refinement but also the governance and implementation contexts in which PRS are deployed (3, 10). Expanding international collaboration beyond existing hubs, aligning funding mechanisms with diverse health priorities, and integrating ethical and population-sensitive considerations into PRS research will be important for supporting responsible and globally informed implementation (14, 27).

### 4.6 Strengths and limitations

This study’s strengths include its comprehensive scope, extended time horizon, and integration of multiple bibliometric dimensions, providing a holistic view of PRS research evolution (17, 18). However, several limitations merit consideration. Reliance on the Web of Science database may underrepresent research published in regional journals, non-English outlets, or emerging interdisciplinary venues (28). Funding acknowledgements reflect presence rather than magnitude of support and do not capture grant size or duration. Importantly, a detailed assessment of ancestry composition in discovery and validation cohorts was beyond the scope of this bibliometric analysis and would require dedicated cohort-level methodological review; therefore, conclusions regarding population representation should be interpreted cautiously. Finally, bibliometric indicators cannot fully assess scientific quality or clinical impact (22). Despite these limitations, the patterns identified offer robust insights into the structural dynamics shaping PRS research.

## 5 Conclusion

Polygenic risk score research has undergone rapid expansion over the past 25 years, evolving from methodological experimentation to a central component of precision medicine research. This bibliometric analysis indicates that PRS scholarship is approaching structural maturity, characterized by high publication volume, thematic consolidation, and increasing clinical orientation. However, the field’s bibliometric footprint remains concentrated, with research output, funding acknowledgements, and institutional leadership dominated by a limited number of high-income countries and research-intensive centers. While bibliometric indicators do not directly reflect the ancestry composition of study populations or the performance of PRS across diverse groups, these concentration patterns highlight the importance of broadening global participation in PRS research and infrastructure development. Continued efforts to support inclusive collaboration, diversified funding ecosystems, and transparent reporting practices will be important for fostering globally informed and clinically robust implementation of PRS in precision medicine.

## Data Availability

The datasets analyzed in this study were obtained from the Web of Science Core Collection database using a predefined search strategy. Access to the database requires a subscription. The full search strategy and extracted bibliometric data are available from the corresponding author upon reasonable request to ensure reproducibility.
