# Supplementary figures and images for: Global trajectories of polygenic risk score research: a systematic bibliometric review of precision medicine, equity, and clinical translation
Source: Front Med (Lausanne). 2026 Apr 7;13:1779659. doi: 10.3389/fmed.2026.1779659 (PMC13096031; doi:10.3389/fmed.2026.1779659)

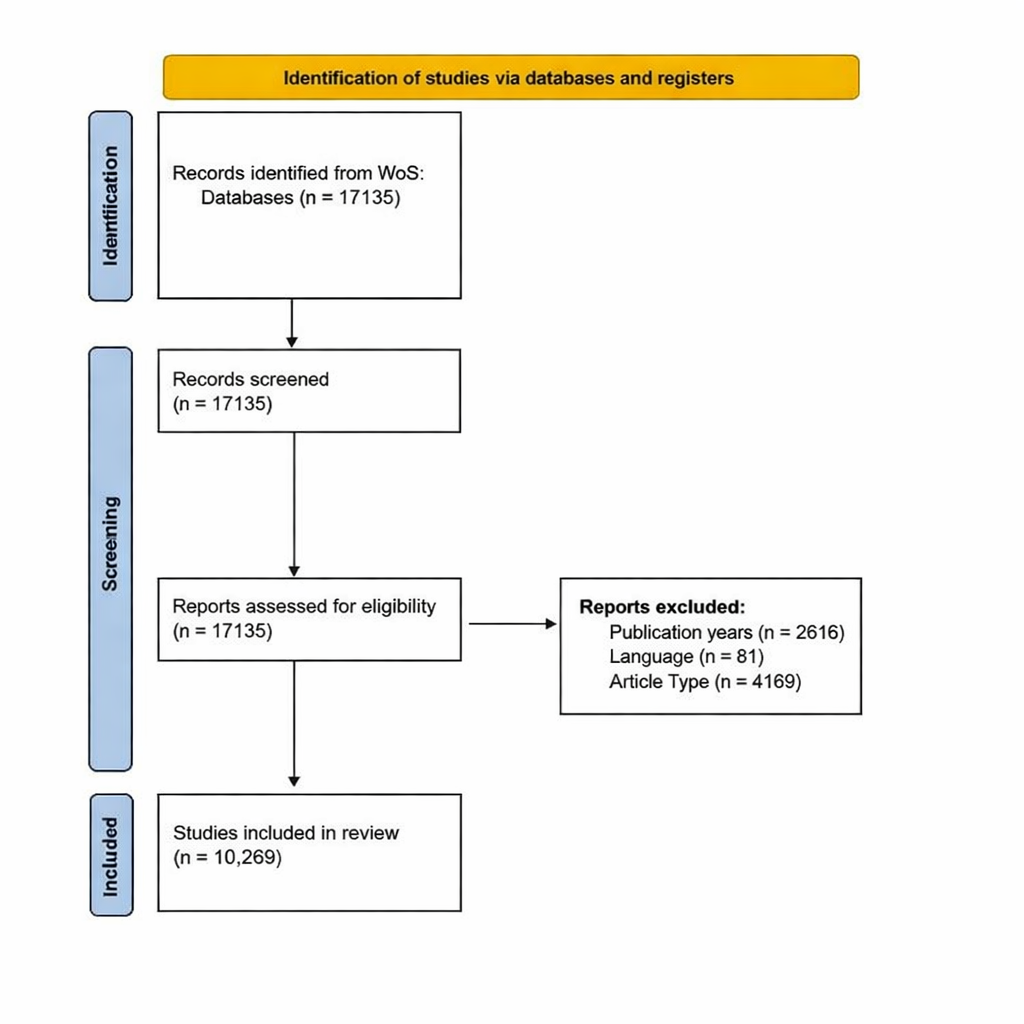

Supplement: Supplementary file 1 [file image_1.png]
